# Methylenetetrahydrofolate Reductase Gene C677T and A1298C Polymorphic Sequence Variations Influences the Susceptibility to Chronic Myeloid Leukemia in Kashmiri Population

**DOI:** 10.3389/fonc.2019.00612

**Published:** 2019-07-24

**Authors:** Shahid M. Baba, Zafar A. Shah, Khushboo Javaid, Arshad A. Pandith, Javeed Rasool, Sajad A. Geelani, Rafia A. Baba, Shajrul Amin, Gul Mohammad

**Affiliations:** ^1^Department of Immunology and Molecular Medicine, Sher-I-Kashmir Institute of Medical Sciences, Srinagar, India; ^2^Department of Clinical Biochemistry, University of Kashmir, Srinagar, India; ^3^Advanced Centre for Human Genetics, Sher-I-Kashmir Institute of Medical Sciences, Srinagar, India; ^4^Department of Clinical Hematology, Sher-I-Kashmir Institute of Medical Sciences, Srinagar, India; ^5^Department of Medical Oncology, Sher-I-Kashmir Institute of Medical Sciences, Srinagar, India

**Keywords:** MTHFR, CML, BCR-ABL, PCR, Kashmir, India, RFLP

## Abstract

**Background:** Methylenetetrahydrofolate reductase (MTHFR) gene is a crucial regulator of folate metabolism and its two prominent polymorphic variants C677T and A1298C lead to decreased MTHFR enzyme activity.

**Aim of the Study:** We planned this case–control study based on numerous studies supporting the association of MTHFR polymorphisms (C677T and A1298C) with CML risk in different ethnic populations. Therefore, the influence of these polymorphisms on CML susceptibility was investigated among Kashmiri population (North India).

**Materials and Methods:** Polymerase chain reaction/restriction fragment length polymorphism (RFLP) technique was employed for genotyping MTHFR C677T and A1298C SNP's in 125 CML patients as against 150 age and gender matched healthy controls.

**Results:** A significant difference was observed in frequency of 677CT genotype between cases and controls [46.4 vs. 27.3% (*p* = 0.0005)]. Similarly combined 677CT+TT genotype showed significant difference between cases and controls [50.4 vs. 28.6% (*p* = 0.0002)]. Both MTHFR 677CT and 677CT+TT genotypes imposed greater than 2-fold risk of developing CML (OR = 2.4, 95%CI: 1.46–4.05; OR = 2.5, 95%CI: 1.53–4.16). In case of A1298C SNP, the frequency of 1298AC genotype was higher in controls (64.0%) as compared to CML cases (48.8%) (*p* = 0.04) and imparted a significant protective role from CML predisposition. Furthermore, haplotype analysis revealed only “677CT/1298AA” haplotype significantly increased the risk of CML predisposition [(*p* = 0.008) (OR = 3.2, 95% CI: 1.3–7.4)].

**Conclusion:** We conclude that both MTHFR C677T and A1298C polymorphisms may be important genetic modifiers and seem to have a plausible role to confer risk of CML in Kashmiri population, where C677T SNP strongly increases the risk of CML while as A1298C SNP has a protective effect.

## Introduction

Chronic Myeloid Leukemia (CML) whose incidence and prevalence has dramatically increased in recent past is the most common myeloid malignancy. CML is a disorder of the hematopoietic stem cell and leads to the buildup of myeloid precursors in bone marrow, peripheral blood and body tissues (1). CML results from t(9;22) that leads to the generation of hybrid fusion gene BCR-ABL1 which generates genomic instability by disrupting the balance between proliferation and apoptosis of cells and drives the leukemic changes in CML (2, 3). Although the t(9;22) is the primary cause for the development of CML, the factors that lead to the induction of Philadelphia chromosome are still unknown. Despite well-documented clinical features and pathology of the disease, very little is known about the individual's susceptibility to CML (3–5). Hereditary or familial associations with the development of CML have not been reported yet.

Association studies have provided evidence which suggests single nucleotide polymorphisms in key genes might increase the risk of CML (6, 7). Therefore, identification of CML susceptibility genes will elucidate disease pathogenesis and provide insights about the risk assessment of the disease. Epidemiological studies indicate an effective association between low folate intake and increased cancer risk. The notion that pathogenesis of CML is linked to the folate metabolism has also been hypothesized (8). Among the diverse group of enzymes that affect the metabolism of folic acid, the most promising role has been attributed to 5,10-methylenetetrahydrofolate reductase (MTHFR).

MTHFR gene in humans is found to be positioned on chromosome one comprising of 11 protein-coding sequences (9). The fate of folate metabolism is regulated by MTHFR catalyzed one-step irreversible reduction of 5, 10-methylenetetrahydrofolate to 5-methylTHF. The latter is found to be a typical form of folate in serum. 5-methylTHF is the major carbon donor for the conversion of homocysteine to methionine, which is later on used for DNA synthesis (10). Weak activity of MTHFR may dampen the 5-methyltetrahydrofolate pathway leading to 5-methyltetrahydrofolate build up that causes reduction in the conversion of dUMP to dTMP. Once dUMP to dTMP methylation is deficient, Uracil can misincorporate into DNA, consequentially resulting in DNA double-stranded breaks that leads to numerous anomalies including hematological malignancies (11, 12).

The decreased functional activity of MTHFR has been attributed to two single gene variants (rs1801133) and (rs1801131). For (rs1801133), the SNP arises due to a missense mutation at nucleotide position 222 in exon 4 of MTHFR gene causing C > T substitution that results in the replacement of alanine with valine. The mutation leads to increased plasma homocysteine via production of MTHFR with lesser enzyme activity at 37°C (13). It has been shown that the carriers of homozygous genotype (MTHFR 677 TT) possess only 30 percent MTHFR enzyme activity in comparison to the wild genotype, whereas 60% enzyme activity is found in individuals with heterozygous (MTHFR 677 CT) genotype (10).

In exon 7, A1298C polymorphism at codon 429 results from the substitution of glutamine with alanine, which results in diminished enzyme activity up to 33% (14). Numerous studies evaluating the possible link between these two MTHFR SNPs in CML have been reported but the findings of these correlation studies are controversial (4, 15).

Since such a possibility has never been investigated in our ethnically conserved population, the current case-control study was attempted to investigate the prominent MTHFR C677T and A1298C SNPs as a measure of risk for CML development in ethnic Kashmiri population.

## Materials and Methods

The present case-control study included 125 CML patients from Kashmir who visited the outpatient clinic or inpatient wards of departments of Medical Oncology and Clinical Hematology, Sher-i-Kashmir Institute of Medical Sciences (SKIMS), J&K (North India).

All patients were assessed clinically and diagnosed by standard methods like blood picture, bone marrow analysis, and detection of Philadelphia chromosome by peripheral blood/bone marrow Cytogenetics. The diagnosis was confirmed by molecular detection of BCR-ABL1 hybrid fusion gene by reverse transcriptase polymerase chain reaction (RT-PCR) and real time polymerase chain reaction (Rq-PCR). Patient details were collected by personal interaction with the patients and laboratory parameters like total leukocyte count (TLC), differential leukocyte count (DLC), and platelet counts were recorded from the routine CBC/bone marrow reports. We further evaluated risk scores like Sokal and EUTOS (European Treatment and Outcome Study) for the CML patients. Sokal and EUTOS scores were calculated from the clinical parameters recorded at diagnosis before start of treatment. Sokal score was determined as per the age, spleen size, platelet count, peripheral blood blasts, and patients were risk stratified as low-risk (Sokal score <0.8), intermediate-risk (Sokal score 0.8–1.2) and high-risk (>1.2) (16). EUTOS score was calculated on the spleen size and percentage of basophils in the peripheral blood. Patients were categorized as low-risk (score <87) and high-risk (score ≥87) (17). We determined the Sokal and EUTOS scores using online formulas on the BloodRef network site (http://www.bloodref.com). The control group comprised of age, sex, and ethnicity matched 150 healthy blood donors with no history of any malignancy and living in the same geographical area as the patients. This study was conducted only after taking informed consent from all participants and approval from the local SKIMS Ethics committee.

### DNA Extraction

Genomic DNA from the peripheral blood/bone marrow leukocytes of CML cases and peripheral blood of control subjects was extracted using QIAamp DNA Blood Mini Kit (Cat No. 51104), Qiagen (Germany) and its concentration and purity was analyzed by Bio spectrophotometer (Eppendorf AG; Serial No: 6137EQ102539; Germany).

### Genotyping of the MTHFR C677T and A1298C SNPs

Genotyping of the two SNPs was performed as described by Wiemels et al. (18). Genomic DNA was amplified in 25 μl reaction volume that contained 2 μl of extracted DNA (100 ng), 0.5 μl of each primer (10 pmol), 0.5 μl dNTPs (10 mmol), 4 μl 10x PCR buffer with MgCl_2_ and sterile water to make up the final volume of 25 μl. For amplification of the MTHFR C677T & 1298C SNPs, primer sets used were forward 5′-TGAAGGAGAAGGTGTCTGCGGGA-3′; reverse 5′-AGGACGGTGCGGTGAGAGTG-3 and forward 5′-GCAAGTCCCCCAAGGAGG-3′;reverse 5′-GGTCCCCACTTGCAGCATC-3′ (Eurofen Genomics, India). DNA was amplified in Agilent SureCycler 8800 thermocycler (USA) by heating the reaction volume at 94°C for 5 min, 94°C for 35 s, 58°C for 35 s, 72°C for 35 s (35 cycles), and 72°C for 7 min. Electrophoresis of 10 μl amplified products on 2% agarose gel displayed 198 and 145 bp amplicons, respectively, for MTHFR C677T & 1298C SNPs.

RFLP analysis was done using endonuclease *Hinf1* (Fermentas, Germany) for C677T and *MobII* (Fermentas, Germany) for A1298C SNPs according to the manufacturer's instructions. Four percent agarose gel was used to resolve the digested products and visualized on Flourchem HD2 gel doc (USA).

For MTHFR C677T SNP, an uncut 198 bp band depicted wild type genotype CC whereas electrophoretic pattern displayed two digested bands (175 and 23 bp) for homozygous variant TT and three bands (198, 175, and 23 bp) for heterozygous CT genotype. For A1298C SNP, the enzyme *MobII* has a single restriction site on mutant CC and two restriction sites on wild-type AA alleles. Hence, three bands of 79, 37, and 29 bp correspond to wild type AA genotype, while mutant CC genotype produced two digested bands of 108 and 37 bp and heterozygous AC genotype produces four bands of 108, 79, 37, and 29 bp.

### Statistics

The significance of the present study was evaluated by Chi-square or Fisher's exact test. Frequency and percentage were used to express qualitative data. Descriptive analyses included frequencies of genotypes and alleles. Odds ratio (OR) as an estimate of relative risk and the 95% confidence interval (CI) was calculated using SPSS version 12 data analysis software. For all analysis, *P*-values at the level of 0.05 were taken as statistically significant.

## Results

This case-control study comprised of 125 CML patients as cases and 150 age and sex matched healthy blood donors with no history of malignancy as controls. The patient group comprised of 66 (52.8%) males and 59 (47.2%) females with age ranging from 8 to 75 years (median 40.5 years). The median age in the control group was 43.0 years (Range 19–58 years). In our study, 88% (110) of the cases were diagnosed as chronic phase CML (CML-CP) patients, while as 10.4% (13) were found in accelerated phase and only 1.6% (02) in blast crisis. Distribution of genotypes of MTHFR-C677T and MTHFR-A1298C polymorphisms were not related to clinical parameters of CML patients like gender, age, dwelling, phase of the disease, and laboratory findings like TLC and Platelet counts (Tables 1, 2).

**Table 1:** Clinical and Laboratory parameters of CML patients according to different MTHFR C677T genotypes.

**Particulars**	**Total** **(*n* = 125)**	**Wild** **(CC = 62)**	**Variant (CT+TT = 63)**	**OR(95%CI)**	***p*-value**
**Gender**					
Male	66 (52.8%)	32 (51.6%)	34 (54.0%)	1.0 (0.52–2.15)	0.9
Female	59 (47.2%)	30 (48.4%)	29 (46.0%)		
**Age**					
<40	52 (41.6%)	29 (46.8%)	23 (36.5%)	0.6 (0.30–1.28)	0.27
≥40	73 (58.4%)	33 (53.2%)	40 (63.5%)		
**Dwelling**					
Rural	106 (84.8%)	52 (83.9%)	54 (85.7%)	1.1 (0.43–3.06)	0.80
Urban	19 (15.2%)	10 (16.1%)	09 (14.3%)		
**TLC count**					
>12,000	87 (69.6%)	43 (69.4%)	44 (69.8%)	1.0 (0.47–2.19)	1.0
≤12,000	38 (30.4%)	19 (30.6%)	19 (30.1%)		
**Plt. count**					
>1Lac	103 (82.4%)	53 (85.5%)	50 (79.4%)	0.6 (0.25–1.66)	0.48
≤1 Lac	22 (17.6%)	09 (14.5%)	13 (20.6%)		
**Disease phase**					
CP	110 (88.0%)	52 (83.9%)	58 (92.1%)		
AP	13 (10.4%)	09 (14.5%)	04 (6.3%)	2.5 (0.72–8.63)	0.15
BC	02 (1.6%)	01 (1.6%)	01 (1.6%)	1.1 (0.6–18.2)	1.0
**Sokal risk groups**					
Low	28 (22.4%)	11 (17.8%)	17 (27.0%)		
Intermediate	68 (54.4%)	39 (62.9%)	29 (46.0%)	–	
High	29 (23.2%)	12 (19.3%)	17 (27.0%)		0.16
**EUTOS score**					
Low Risk	89 (71.2%)	47 (75.8%)	42 (66.7%)		
High Risk	36 (28.8%)	15 (24.2%)	21 (33.3%)	–	0.34

**Table 2:** Clinical and Laboratory parameters of CML patients according to different MTHFR A1298C genotypes.

**Particulars**	**Total** **(*n* = 125)**	**Wild** **(AA = 54)**	**Heterozygous** **(AC+CC = 71)**	**OR** **(95%CI)**	***p*-value**
**Gender**					
Male	66 (52.8%)	30 (55.6%)	36 (50.7%)	0.82 (0.40–1.67)	0.71
Female	59 (47.2%)	24 (44.4%)	35 (49.3%)		
**Age**					
<40	52 (41.6%)	25 (46.3%)	27 (38.0%)	0.7 (0.34–1.45)	0.36
≥40	73 (58.4%)	29 (53.7%)	44 (62.0%)		
**Dwelling**					
Rural	106 (84.8%)	49 (90.7%)	57 (80.3%)	0.41 (0.13–1.23)	0.13
Urban	19 (15.2%)	05 (9.3%)	14 (19.7%)		
**TLC count**					
>12,000	87 (69.6%)	37 (68.5%)	50 (70.4%)	1.09 (0.50–2.35)	0.84
≤12,000	38 (30.4%)	17 (31.5%)	21 (29.6%)		
**Plt. count**					
>1Lac	103 (82.4%)	41 (75.9%)	62 (87.3%)	2.18 (0.85–2.27)	0.15
≤1Lac	22 (17.6%)	13 (24.1%)	09 (12.7%)		
**Disease phase**					
CP	110 (88.0%)	50 (92.6%)	60 (84.5%)		
AP	13 (10.4%)	04 (7.4%)	09 (12.7%)	0.53 (0.15–1.83)	0.38
BC	02 (1.6%)	00 (0.0%)	02 (2.8%)	0.39 (0.04–3.95)	0.62
**Sokal risk groups**					
Low	28 (22.4%)	13 (24.1%)	15 (21.1%)		
Intermediate	68 (54.4%)	30 (55.5%)	38 (53.5%)	–	0.79
High	29 (23.2%)	11 (20.4%)	18 (25.4%)		
**EUTOS score**					
Low risk	89 (71.2%)	36 (66.7%)	53 (74.6%)		
High risk	36 (28.8%)	18 (33.3%)	18 (25.4%)	–	0.42

In the current study, as per the Sokal score, 28 (22.4%) patients were stratified as low risk, 68 (54.4%) as intermediate risk and 29 (32.2%) as high risk group patients. According to the EUTOS score, 89 (71.2%) patients were categorized as low risk and 36 (28.8%) as high risk patients. Genotypic distribution of the two MTHFR SNPs did not relate either with the Sokal risk groups (*p* = 0.16 and *p* = 0.79, respectively) or EUTOS score (*p* = 0.34 and *p* = 0.42, respectively) (Tables 1, 2).

Genotypes, combined genotypes, haplotypes and allelic frequency of MTHFR gene C677T and A1298C SNPs are given in (Tables 3, 4).

**Table 3:** Genotypes distribution and allelic frequencies of the MTHFR SNPs in CML patients and controls.

**Polymorphism**	**CML cases** ***n* = 125 (%)**	**Controls** ***n* = 150 (%)**	**OR(95%CI)**	***p*-value**
**MTHFR C677T**				
CC	62 (49.6%)	107 (71.4%)	1 (Ref)	
CT	58 (46.4%)	41 (27.3%)	2.4 (1.46–4.05)	**0.0005**
TT	05 (4.0%)	02 (1.3%)	4.3 (0.81–22.90)	0.10
CT+TT	63 (50.4%)	43 (28.6%)	2.5 (1.53–4.16)	**0.0002**
Frequency of C	182 (72.8%)	255 (85.0%)	1 (Ref)	
Frequency of T	68 (27.2%)	45 (15.0%)	2.1 (1.38–3.22)	**0.0004**
**MTHFR A1298C**				
AA	54 (43.2%)	49 (32.7%)	1 (Ref)	
AC	61 (48.8%)	96 (64.0%)	0.57 (0.34–0.95)	**0.04**
CC	10 (8.0%)	05 (3.3%)	1.8 (0.57–5.68)	0.40
AC+CC	71 (56.8%)	101 (67.3%)	0.6 (0.39–1.04)	0.08
Frequency of A	169 (67.6%)	194 (64.7%)	1 (Ref)	
Frequency of C	81 (32.4%)	106 (35.3%)	0.7 (0.5–1.07)	0.13

Values in bold are statistically significant values.

**Table 4:** Combined haplotypes analysis of MTHFR SNPs in CML patients and controls.

**MTHFR gene**		**Control group** **(n = 150)**	**CML patients** **(n = 125)**	**OR** **(95 % CI)**	**P-value**
**C677T**	**A1298C**				
CC	AA	36 (24.0%)	24 (19.2%)	1(Ref)	
CC	AC	68 (45.4%)	33 (26.4%)	0.7 (0.3–1.4)	0.3
CC	CC	03 (2.0%)	05 (4.0%)	2.5 (0.5–11.4)	0.2
CT	AA	13 (8.7%)	28 (22.4%)	3.2 (1.3–7.4)	**0.008**
CT	AC	26 (17.3%)	27 (21.6%)	1.5 (0.7–3.2)	0.2
CT	CC	02 (1.3%)	03 (2.4%)	2.2 (0.3–14.4)	0.6
TT	AA	00 (0.0%)	02 (1.6%)	4.4 (0.4–45.1)	0.3
TT	AC	02 (1.3%)	01 (0.8%)	0.7 (0.06–8.7)	1
TT	CC	00 (0.0%)	02 (1.6%)	4.4 (0.4–45.1)	0.3

*Values in bold are statistically significant values*.

For MTHFR C677T polymorphism, the respective genotype frequencies of CC, CT, and TT were 49.6, 46.4, and 4.0% in CML patients and 71.4, 27.3, and 1.3% in controls. CC genotype frequency was more in healthy control subjects 71.4% in comparison to cases 49.6%. A significant difference was observed in frequency of heterozygous genotype 677CT 46.4 vs. 27.3% (*p* = 0.0005) (OR = 2.4, 95% CI: 1.46–4.05) and combined variant genotype (677CT+TT) 50.4 vs. 28.6% (*p* = 0.0002) (OR = 2.5, 95% CI: 1.53–4.16) between patients and controls (Table 3). MTHFR 677T allele frequency was 27.2% in CML cases and showed a significant difference compared to controls 15.0% (*p* = 0.0004) (OR = 2.1, 95% CI: 1.38–3.22) (Table 3).

For MTHFR A1298C SNP, the frequencies of AA, CC, and AC genotypes was 43.2, 8.0, and 48.8% in cases and 32.7, 3.3, and 64.0% in controls, respectively. In addition, the combined variant genotypes (1298 AC+CC) frequency in cases was 56.8% with no significant differences when compared to controls (67.3%) (*p* = 0.08) (OR = 0.6, 95% CI: 0.39–1.04). However, a significant difference was observed in the frequency of 1298AC genotype among CML cases 64.0% and controls 48.8% (*p* = 0.04) (OR = 1.8, 95% CI: 0.57–5.68) (Table 3). Further, the frequency of C allele was comparable between CML cases (32.4%) and controls (35.3%) suggesting no significant differences between the groups for the 1298 C allele (*p* = 0.13) (OR = 0.7, 95% CI: 0.5–1.07) (Table 3).

In the haplotype analysis, only 677CT/1298AA haplotype showed significant association with increased CML risk (*p* = 0.008) (OR = 3.2, 95% CI: 1.3–7.4) (Table 4).

## Discussion

CML, which arises due to genetic damages to pluripotent hematopoietic stem cells, has peculiar clinical and biological features (19). Although information about the factors which predispose individuals to CML is scanty (3), but its risk may be increased due to quantitative and qualitative changes in folate metabolism caused by these genetic damages (20).

Folate acts as a transporter of carbon moiety which is used for converting homocysteine to methionine and also for the production of purine and pyrimidine (21). MTHFR is one of the main regulators of folate metabolism and polymorphic variations like C677T and A1298C in this gene leads to variant alleles which results in decreased MTHFR activity *in vitro* in comparison to the wild-type allele and thereby increases CML susceptibility (22, 23).

Keeping in view the conserved gene pool of our ethnic population due to the common practice of interrelated marriages, the pattern of polymorphic variations always remains an important issue to evaluate, for identification of individuals with increased disease susceptibility. Therefore, this case-control study is the first from our region that aims to observe whether sequence polymorphic variations at C677T and A1298C in MTHFR gene predisposes individuals to CML in our population (Kashmiri, North India). Studies in varying ethnic groups regarding the possible association between MTHFR polymorphic sequence variations and susceptibility to CML have been carried out but the reported results are conflicting (Table 5).

**Table 5:** Significance of methylenetetrahydrofolate reductase (MTHFR) C667T and A1298C polymorphisms in different ethnic populations.

**Author**	**Country**	**SNP's studied**	**Cases** **(*n* =)**	**Controls** **(*n* =)**	**Association**
Deligezer et al. (37)	Turkey	*C677T*	132	161	Insignificant
Hur et al. (30)	Korea	*C677T, A1298C*	40	200	Significant
Moon et al. (28)	Korea	*C677T, A1298C*	115	434	Significant
Barbosa et al. (15)	Brazil	*C677T, A1298C*	67	100	Insignificant
Kim et al. (29)	Korea	*C677T, A1298C*	149	1,700	Insignificant
Ismail et al. (25)	Jordan	*C677T, A1298C*	149	170	Significant
Vahid et al. (5)	Iran	*C677T, A1298C*	38	97	Insignificant
Jankovic et al. (34)	Serbia	*C677T*	43	26	Insignificant
Hussain et al. (26)	India	*C677T*	43	251	Significant
Lordelo et al. (3)	Brazil	*C677T, A1298C*	41	155	Significant
Jakovljevic et al. (35)	Serbia	*C677T*	52	53	Insignificant
Khorshied et al. (24)	Egypt	*C677T, A1298C*	97	130	Insignificant
Rabab et al. (36)	Egypt	*C677T, A1298C*	85	100	Significant
Banescu et al. (27)	Romania	*C677T, A1298C*	151	305	Significant
Dorgham et al. (31)	Algeria	*C677T, A1298C*	90	100	Significant
Hairong et al. (33)	–	*C677T, A1298C*	Meta-Analysis		Significant
Li et al. (32)	–	*C677T, A1298C*	Meta-Analysis		Significant
Present study	India	*C677T, A1298C*	125	150	Significant

In this study, the frequencies of MTHFR C677T variant genotypes CT and TT in CML patients were 46.4 and 4.0%, respectively, which was higher when compared to controls. The frequency of MTHFR C677T genotypes found was almost similar to that reported by other authors from different ethnic regions (3, 24, 25). The variation in genotype frequencies can result from geographic and ethnic differences. However, higher frequencies of TT genotypes were reported in Iran by Vahid et al. and in India by Hussain et al., but the number of cases in these studies was comparatively less (5, 26).

In our series of CML patients, the presence of variant genotype 677CT was related to a significant 2.4-fold increased CML susceptibility (OR = 2.4, 95% CI: 1.46–4.05) (*p* = 0.0005) using 677 CC as reference. Nearly same 2.4 fold increased risk of CML was observed for combined variant genotype MTHFR CT+TT when compared with CC genotype (OR = 2.5, 95% CI: 1.53–4.16) (*p* = 0.0002) (Table 3). A recent study conducted by Banescu et al., from Romania reported a significant 1.55 and 1.89-fold increased CML risk associated with 677 CT and TT variant genotypes, respectively (27). The results of our study are further augmented by likewise studies conducted by Ismail et al. and Hussain et al. who reported 4.5 and 2.8-fold increased CML risk in patients having the homozygous genotype TT (25, 26).

We observed a higher 677T allele frequency (27.2%) in our CML cases which depict that 677T allele significantly influences the risk of CML in Kashmiri population (*p* = 0.0004). In likewise studies by Hussain et al. and Banescu et al., the authors also reported a significant impact of the 677T allele on risk of CML in patients from North Indian and Romania (26, 27). However, a study conducted by Moon et al. reported no significant influence of MTHFR 677T allele on CML risk in Korean population (28). These observations depict a strong association between MTHFR C677T SNP and predisposition to CML and it could be considered as a risk factor for CML development in our population. However, there are certain studies where no significant association of the same SNP was reported with the risk of CML development (3, 28, 29).

With respect to MTHFR A1298C polymorphism, the frequency of AC and CC genotypes in cases was 48.8 and 8.0%, respectively, which was close to that reported by Lordelo et al. and Khorshied et al. (3, 24). Interestingly we reported higher frequencies of the heterozygous genotype AC in controls than in CML cases (64 vs. 48.8%). A similar scenario was reported by Vahid et al. and Ismail et al. (5, 25). Furthermore, we reported a significant 1.7-fold decrease in CML risk in cases with 1298AC genotypes when compared with 1298 AA genotype (OR = 0.57, 95% CI: 034–0.95), (*p* = 0.04) (Table 3). Hur et al. also reported 2.5–2.9 folds increased protective role of 1298AC genotype in Korean CML patients (30). Lordelo et al. showed that the AC genotype of the MTHFR 1298 A>C polymorphism significantly decreased the risk of CML, particularly in the case of young adult females (*p* = 0.047) (3). Further study by Robin et al. reported a stronger association between 1298AC genotype and decreased relapse risk in CML patients after hematopoietic cell transplant (4). Therefore, together these reports suggest that 1298 polymorphic variants may have a protective outcome in CML development. However, studies by Moon et al. and Dorgham et al. reported 5 and 2-fold increased CML risk attributed to this genetic polymorphism (28, 31). Also meta-analysis by Li et al. and Hairong et al. reported an increased CML risk for 1298C variant carriers (32, 33). This variability in different ethnic populations implies that the functional impact of these SNP's on CML susceptibility is a population dependent phenomenon.

In the present study, we found 677CT/1298AA haplotype associated with more than 3-fold increased risk of developing CML as its frequency was significantly higher in cases than controls (*P* = 0.008). However, Banescu et al. reported 677CT/1298AC compound heterozygote as a risk factor for developing CML in Romanian population (27).

Various clinicopathological characteristics including, disease phase, Sokal, and EUTOS score in patients with CML were stratified but did not show any correlation with MTHFR 677 C > T and 1298 A > C.

In summary, the main findings of the study emphasize the importance of MTHFR C677T variant genotypes for conferring strong risk to CML. On the other hand, MTHFR A1298C heterozygous genotype selectively is shown to exert a protective impact for CML cases as is reported in several studies. Although the present report had a limitation of relatively small study population, but the findings therein revealed that the incidences of CML are more dependent on folate status. We conclude that both MTHFR C677T and A1298C SNPs may be important genetic modifier and seem to have a plausible role to confer risk of CML in Kashmiri (North Indian) population. The apparent inconsistencies between our results and those of previous studies, in different populations, may be due to the diverse number of patients studied, different genotype frequencies, different ethnicities, and the absence of information on dietary folate intake.

Additional studies, with a larger sample size in various geographical areas are augmented to explore MTHFR C677T and A1298C SNPs in CML pathogenesis. Further the dual role of the two SNPs in CML seems to have a promise for functional analysis to analyze the leukemogenesis of chronic myeloid leukemia.

## Data Availability

The raw data supporting the conclusions of this manuscript will be made available by the authors, without undue reservation, to any qualified researcher.

## Ethics Statement

This study was carried out in accordance with the recommendations of the Code of Ethics of the World Medical Association. All subjects gave written informed consent in accordance with the Declaration of Helsinki. The protocol was approved by the Sher-i-Kashmir Institute of Medical Sciences Ethics committee.

## Author Contributions

SB conceptualized, designed and supervised the study, did lab work, and also drafted the manuscript. ZS supervised the study, provided the consumables and logistical support, and submitted the manuscript. KJ performed the lab work. AP did proofreading and corrected the manuscript. JR, SG, and GM provided patient samples. RB performed lab work and drafted the manuscript. SA did compilation and statistical work.

### Conflict of Interest Statement

The authors declare that the research was conducted in the absence of any commercial or financial relationships that could be construed as a potential conflict of interest.
